# Syngas Production over Nanosized Multicomponent Co-Fe-Containing Catalysts

**DOI:** 10.3390/nano15231814

**Published:** 2025-11-30

**Authors:** Kuralay T. Tilegen, Sholpan S. Itkulova, Makpal A. Zhumash, Yerzhan A. Boleubayev, Arlan Z. Abilmagzhanov

**Affiliations:** 1 D.V. Sokolsky Institute of Fuel, Catalysis and Electrochemistry, 142, Kunaev Str., Almaty 050010, Kazakhstan; kuralay.tilegen@alumni.nu.edu.kz (K.T.T.); makpal.zhumash@mail.ru (M.A.Z.); e.boleubaev@ifce.kz (Y.A.B.); a.abilmagzhanov@ifce.kz (A.Z.A.); 2 School of Chemical Engineering, Kazakh British Technical University, Tole Bi Street, 59, Almaty 050000, Kazakhstan

**Keywords:** Co-Fe nanosized catalysts, carbon dioxide, methane, syngas, synergetic effect

## Abstract

Carbon dioxide reforming of methane is a promising technology to recycle and reduce greenhouse gases (CH_4_, CO_2_) into valuable chemicals and fuels. The Co-Fe catalysts modified with a small amount of Pt and supported on alumina were designed to be explored in dry reforming (DRM) and combined CO_2_-steam reforming (bireforming, BRM) of methane to produce syngas. The catalysts were characterized by physico-chemical methods (i.e., BET, XRD, TEM, SEM, and TPR-H_2_). The synthesized catalysts are the X-ray amorphous nanosized materials with particle sizes of less than 30 nm. The processes were carried out using a feed of CH_4_/CO_2_/H_2_O = 1/1/0–0.5 at varying temperature (400–800 °C) at atmospheric pressure and GHSV = 1000 h^−1^. The combination of Co and Fe in varying ratios with Pt allowed for high activity and selectivity to be maintained. Extents of methane and CO_2_ conversion are varied within a range of 79.5–97.5 and 64.2–85.2%, respectively, at 700–800 °C, while the H_2_/CO ratio in the resulting syngas ranged from 0.98 to 1.30, depending on the catalyst and feed composition. Stability tests conducted for up to 80 h on stream showed no loss of activity of the 10%Co-Fe-Pt/Al_2_O_3_ catalysts in BRM. We believe that high activity of the synthesized catalysts occurs due to synergy in the Co-Fe-Pt system.

## 1. Introduction

There is a global shift toward renewable and sustainable energy sources driven by the depletion of fossil fuels and environmental concerns. In response to rising emissions, carbon capture, utilization, and storage (CCUS) technologies have gained growing attention. Methane, the main component of natural gas, is the second most potent greenhouse gas after CO_2_, with a much higher global warming potential despite its lower concentration. Catalytic methane conversion processes, such as dry reforming and bireforming, offer an efficient route for greenhouse gas utilization and CCUS advancement. To enable large-scale implementation, catalysts with high activity and economic feasibility must be developed [1].

Dry reforming of methane (DRM) and bireforming of methane (BRM or combined steam-dry reforming of methane) are promising technologies that recycle and reduce greenhouse gases (CH_4_, CO_2_) into valuable chemicals and fuels. DRM is extensively studied in the literature due to its scientific, technological, and industrial importance, and involves the production of hydrogen and carbon monoxide as primary reaction products. DRM, expressed in Equation (1), produces highly pure syngas with a ratio of H_2_/CO equal or less than 1. Syngas, when balanced with the right proportion of hydrogen to carbon dioxide, can serve as a valuable intermediary in methanol, dimethyl ether production, and hydroformylation processes [2]. Hydrogen, a product of DRM (Equation (1)), is a versatile fuel with the potential to replace fossil fuels in various sectors, and is thus highlighted as particularly promising.

CH_4_ + CO_2_ ↔ 2H_2_ + 2CO   ΔH = 247 kJ/mol (1)

However, carbonaceous deposits on catalysts pose a significant challenge, reducing catalyst activity and blocking pores. It is crucial to adjust parameters to extend catalyst lifespan and enhance hydrogen production efficiency, potentially for industrial use. Modifications such as incorporating a second metal or selecting appropriate support materials are necessary. Emphasizing catalysts operable at low temperatures is vital, not only to mitigate coke deposition but also for safer and more economical reaction handling [3,4,5].

Many researchers worldwide are working diligently to improve the catalytic performance of catalysts, focusing particularly on enhancing their stability and activity. Catalyst efficiency is affected by various factors, such as the addition of noble metals to the transition metal catalysts, the selection of support material, the incorporation of promoters, catalyst thermal pre-treatment, and process conditions [6,7,8]. The noble metals exhibit the best catalytic activity and stability in DRM in the order Rh > Ru > Ir > Pt > Pd [9,10]. Ni-based catalysts showed good activity and economic viability [8]. Co-based catalysts have better resistance to coke formation than Ni catalysts, despite reports that they are less active in DRM [11]. The introduction of small amounts of noble metals into supported cobalt catalysts allows for a synergistic effect between Co and noble metals, which facilitates the reduction of cobalt oxides and slows down the temperature decrease, increasing the resistance to carbon deposition and sintering, thereby improving the overall performance of the catalyst.

Although iron is not an extensively studied transition metal in DRM due to its low activity, it is a promising cost-effective promoter with a positive effect on enhancing catalyst activity [12]. Table 1 highlights for comparison the characteristics of some Fe-containing catalysts in DRM found in the literature [12,13,14,15,16,17,18,19,20]. The effect of Fe loading on catalytic performance of Fe-HMS (iron Hexagonal Mesoporous Silicate) catalysts was studied by [21]. It was observed that increasing Fe content in the catalyst resulted in formulation of more synthesis gas. As reported in [12], 75Ni-25Fe/Al_2_O_3_ catalyst demonstrated enhanced catalytic activity and reduced deactivation, attributed to better resistance to coke deposition resulting from the modification of Ni’s electronic properties by Fe. It was established that the best catalytic activity in terms of high H_2_ (86–87%) and CO (85–88%) yields was shown by 5Ni3Fe/10ZrAl catalyst in 420 min of TOS (Time on Stream) due to an increase in reducible NiO and iron species, enhanced CO_2_ interaction over the catalyst surface, and lower coke formation [22].

Among the iron-modified 5Co/Al_2_O_3_ catalysts with varying iron loadings, the optimal iron content was found to be 0.8 wt% due to an improved hydrogen yield of nearly 45% and an H_2_/CO ratio exceeding 0.65. This enhancement is attributed to a decrease in Co_3_O_4_ particle size and a reduction in temperature, along with increased basicity resulting from Fe addition [13].

In combining Fe-Ni catalysts, Fe enhances stability and coke resistance by slowing carbon deposition and promoting coke gasification, which prevents catalyst deactivation. Fe segregates and forms FeO_x_, which can react with surface carbon, helping to remove it and improve the catalyst’s lifetime [23].

In our previous study we examined cobalt catalysts with a small amount of platinum supported on Al_2_O_3_–ZrO_2_ in dry reforming of methane and bireforming. The 5 wt% Co–Pt (95:5)/Al_2_O_3_–ZrO_2_ catalyst exhibited high stability with no sintering or coke formation during 100 h activity tests [24]. In [25], the effect of Pt additives to mesoporous alumina promoted with metal oxides like MgO, ZrO_2_, CeO_2_, and La_2_O_3_ was evaluated and good catalyst stability was reported under DRM conditions. The Pt/CeO_2_-Al_2_O_3_ catalysts exhibited the highest activity for DRM at 700 °C with the conversion results of X(CH_4_) = 90%, X(CO_2_) = 78%, and a high H_2_/CO ratio of 0.90. The catalytic performance was maintained for more than 24 h despite the formation of coke deposits.

Thus, the literature review demonstrates the following:

- Co-based catalysts exhibit activity in DRM, although they are not as widely studied as nickel, and are characterized by greater resistance to coke formation [26,27].
- Platinum enhances coke resistance and can be used in small amounts to improve catalyst stability [27,28].
- Iron has been studied less extensively in DRM due to its relatively low catalytic performance. However, iron-based catalysts possess several advantages: they are resistant to coke formation at high temperatures, less expensive than other metals, and operate efficiently across a wide temperature range. In addition, the redox properties of iron compounds can enhance reducibility, further improving overall catalytic efficiency. That is why iron can be considered a promising and economically efficient promoter that positively influences coke resistance.

In this study, the new catalysts on a base Co and Fe with a small additive of Pt supported on alumina have been prepared, characterized, and tested in a dry and combined CO_2_-steam reaction (bireforming) of methane.

## 2. Materials and Methods

Polymetallic catalysts containing Co, Fe, and Pt were prepared by wet co-impregnation of alumina with aqueous solutions of the corresponding metal precursors: Co(NO_3_)_2_•6H_2_O, Fe(NO_3_)_3_•9H_2_O, and H_2_PtCl_6_•nH_2_O, and purity is 99% for all compounds. The support is γ-alumina granules in the form of balls with a diameter of 3–5 mm and a BET surface area of 140 m^2^/g (Novosibirsk, Russia, purity—99%). After drying, the catalysts were calcined at 400 °C for 3 h and then, prior to testing, were reduced by hydrogen at 400 °C for 1–3 h. The total amount of metals was 10 wt%. The Co/Fe ratio varied and was 7:3, 5:5, and 3:7. The amount of Pt was the same for all three catalysts and was equal to 0.2 wt%. This value corresponds to the composition of 9.8%Co-Fe(7:3)-0.2%Pt/Al_2_O_3_, 9.8%Co-Fe(5:5)-0.2%Pt/Al_2_O_3_, and 9.8%Co-Fe(3:7)-0.2%Pt/Al_2_O_3_ respectively. Element analysis provided by means of the scan electron microscope JSM 6610 LV (JEOL Ltd., Tokyo, Japan) showed a correlation between nominal and actual weight and deviation of no more than 2% on average for each element involved.

The synthesized catalyst was tested during the carbon dioxide reforming (DRM) and bireforming (BRM, when steam was added to CH_4_-CO_2_ feed) of methane. The DRM and BRM processes were carried out in a quartz flow reactor under atmospheric pressure; the CH_4_/CO_2_ ratio was 1:1, the gas hourly space velocity (GHSV) was 1000 h^–1^, and temperatures varied within a range of 400–800 °C. Volume of steam added to a feed of CH_4_/CO_2_ = 1:1 was 20 vol.%, corresponding to a volume ratio of CH_4_/CO_2_/H_2_O = 1:1:0.5. The initial and final reaction products were analyzed online using gas chromatography (GC).

The catalyst was characterized by using transmission electron microscopy (TEM), scanning electron microscopy (SEM), BET (Brunauer-Emmett-Teller), X-ray diffraction (XRD), and H_2_-TPR (hydrogen-Temperature Programmed Reduction) methods.

Physico-chemical analyses of the catalysts were undertaken prior to and after the reaction to understand the effects of the reaction feed and process conditions on the catalyst characteristics (i.e., specific surface area, reducibility of Co, particle morphology and size, element distribution, etc.).

The specific surface areas and average pore diameters were measured using the BET and BJH methods, respectively, with the help of a Thermo Scientific Surfer Gas Adsorption Porosimeter (Thermo Fisher Scientific, Italy) and the Advanced Data Processing program (Version 6.2). For sample preparation, the SURFER instrument is equipped with a GEFRAN 800P (Thermo Fisher Scientific, Italy) degassing unit. The sample was degassed by gradually increasing the temperature from 25 to 220 °C and holding at 220 °C for 120 minutes. The analysis consists of two parts: dead-space measurement with helium gas and adsorption–desorption measurements of the sample using nitrogen gas.

X-ray diffraction (XRD) measurements were performed with the fresh and used catalysts using the CuK_α_ or CoK_α_ radiation of a “Dron-4” powder diffractometer (Bourevestnik, Saint Petersburg, Russia) with CuK_α_ radiation.

To determine the reducibility of metals, the temperature-programmed reduction (H_2_-TPR) was provided. TPR measurements were performed on SETARAM Instrumentation (Caluire, France) using a thermal conductivity detector (TCD) and a 5%H_2_/N_2_ mixture at a flow rate of 20 cm^3^/min. The catalyst samples were heated from ambient temperature to 900 °C with a heating rate of 5 °C/min.

Electron microscopy studies were provided with a JEM-100CX unit. Phase identification was performed with the help of the ASTM standards (American Society for Testing and Materials, Powder Diffraction File. International Centre for Diffraction Data: Swarthmore, PA, USA, 2004).

Scanning electron microscopy (SEM) images were taken using a Jeol JSM 6610 LV instrument using a secondary electron detector. The external surface of the entire catalyst granule as well as the inner surface of a catalyst granule divided in half have been scanned.

## 3. Results and Discussion

### 3.1. Catalyst Characterization

The BET specific surface areas were determined for the ‘fresh’ (before reaction) and ‘spent’ or used (after reaction) samples of the Co-Fe catalysts. For fresh samples, the specific surface area ranges from 168.3 to 190.8 m^2^/g (Table 2). The higher surface area of 190.8 m^2^/g corresponds to a higher Fe content, where the ratio of Co/Fe is 3:7. The specific surface area of all the studied spent catalysts in the DRM and BRM processes decreased notably, ranging from 60.6 to 111.7 m^2^/g. This is due to the potential accumulation of coke on the catalyst surface and its partial blocking of pores. This can substantially reduce the active surface area and lead to a decrease in catalytic activity. In fact, no significant decrease in catalyst activity was observed after 80 h of operation on stream. It should be noted that the spent samples were not subjected to post-reaction treatment; therefore, intermediate species formed during the reaction can remain on the surface of the catalyst, thereby reducing the surface area. For the same reason, there is some increase in the average pore diameter in the samples spent (Table 2).

The elemental composition (in normalized weight percent) of the fresh and spent catalysts obtained by Energy Dispersive X-ray is presented in Table 3. The main elements are O, Al (support—Al_2_O_3_), Fe, and Co (active metals). The nominal metal loadings are 2.9–6.9% Co, 2.9–6.9% Fe, and 0.2% Pt (by mass). The composition of the initial samples is quite close to the nominal value. Pt was not detected, likely due to its low concentration (0.2% of the total mass), which may be below the detection limit. Nevertheless, the improvement of catalytic behavior confirms its presence and effect. The surface elemental composition of the spent catalyst determined by SEM–EDX showed higher Fe and Co contents than the nominal values calculated. This discrepancy likely arises from surface enrichment with the metals under the reaction medium, which promotes migration of Co and Fe species toward the external surface of alumina. As SEM–EDX is a surface-sensitive technique, it reflects the surface rather than the bulk composition, and local heterogeneities or metal agglomerates can further increase the apparent surface concentration.

**Figure 1:**
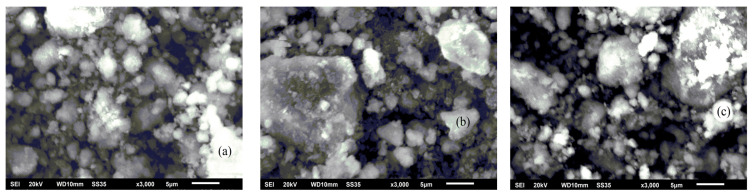
SEM images of the 9.8%Co-Fe(5:5)-0.2%Pt/Al_2_O_3_ catalyst when (**a**) fresh, (**b**) spent in DRM, and (**c**) spent in BRM.

No changes in the catalyst morphology were observed by SEM. Figure 1, for example, shows a typical SEM image of the 9.8%Co-Fe(5:5)-0.2%Pt/Al_2_O_3_ catalyst. The SEM patterns of the catalysts after treatment in DRM (Figure 1b) and BRM (Figure 1c) are similar to those of the fresh sample (Figure 1a).

**Figure 2:**
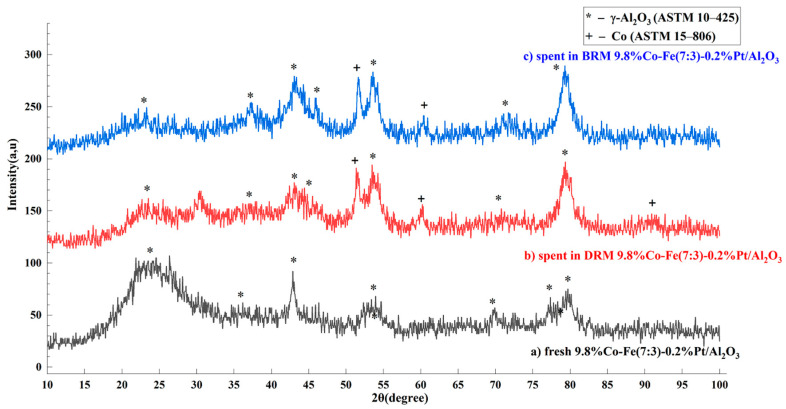
XRD patterns of the 9.8%Co-Fe(7:3)-0.2%Pt/Al_2_O_3_ catalyst samples: fresh and spent in DRM and BRM.

The catalysts before and after the reaction were examined by X-ray diffraction (XRD) to determine their crystal structure and phase composition. No phases other than alumina (JCPDS, 10–425) were detected in fresh samples. In catalysts with a high Co content (Co/Fe ratio = 7:3 and 5:5) after operation in DRM and BRM, only the Co phase (JCPDS, 15–806) was detected. The catalysts are X-ray amorphous due to their high dispersion. Typical X-ray diffraction patterns are shown in Figure 2 for the 9.8%Co-Fe(7:3)-0.2%Pt/Al_2_O_3_ catalyst.

**Figure 3:**
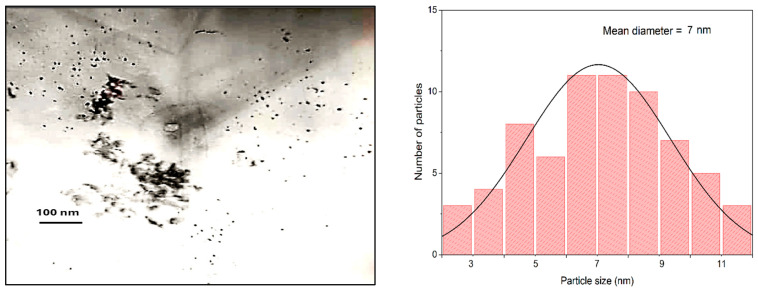
TEM image of 9.8%Co-Fe(5:5)-0.2%Pt/Al_2_O_3_ catalyst.

The TEM study confirms that the synthesized catalysts are the nanomaterials. In the fresh samples, the average size of spherical particles varies within a range of 5–10 nm (Figure 3).

The phases identified by microdiffraction in the fresh sample are attributed to CoO (ASTM, 9–402), Co (ASTM, 5–727), γ-Fe_2_O_3_ (ASTM, 24–81). The samples spent are X-ray amorphous that makes it difficult to identify the X-ray reflexes.

**Figure 4:**
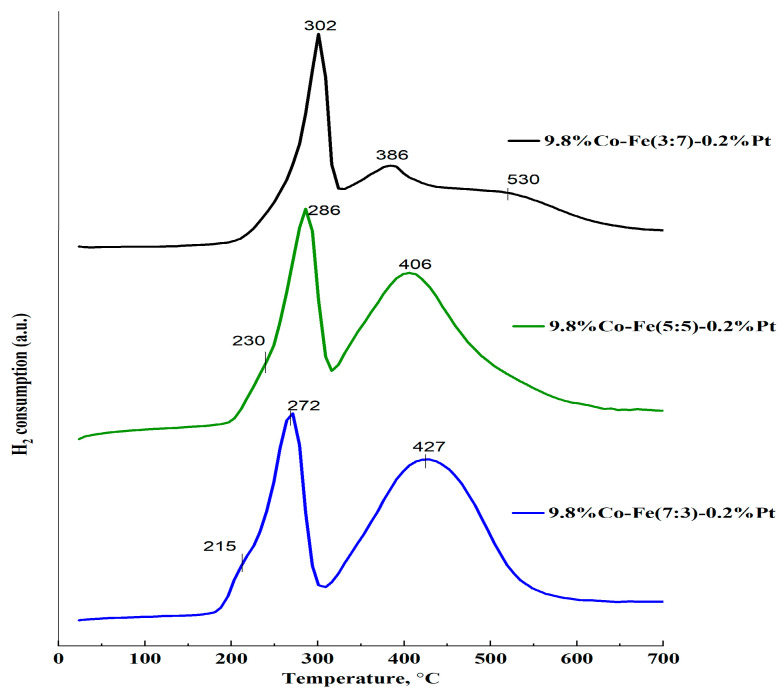
TPR profiles of 10%Co-Fe-Pt/Al_2_O_3_ catalysts.

The TPR profiles of the trimetallic catalysts with the same amount of Pt and variable ratio of Co/Fe are generally similar, with the exception of the sample with a higher Fe content, Co/Fe = 3:7 (Figure 4). In the range of 200–600 °C, two intense peaks were observed for all three catalysts, corresponding to the stepwise reduction of iron [29] and cobalt oxides [30,31].

Based on a previous study conducted by us [32], in our H2-TPR study of 5% Co-Rh/Al_2_O_3_ catalysts with almost the same amount of noble metal Rh (0.25 wt%), two intensive peaks were observed: a low temperature peak at 258 °C, caused by the reduction of Co_3_O_4_ to CoO, and a broad peak at 420 °C, caused by the reduction of CoO to Co^0^. The same pattern was observed for the 5%Co-Pd/Al_2_O_3_ catalysts with 0.25 wt% Pd: two peaks at 230 and 410 °C [33]. Preliminarily, by analogy for the Co-Fe-Pt catalysts, the two-step reduction of Co oxides (Equation (2)) may be proposed.

Co_3_O_4_ → CoO → Co^0^ (2)

Regarding Fe oxides, it is known that generally Fe oxides are reduced at high temperatures, and the reduction of iron oxides is a multistage and stepwise process. If the reduction temperature is lower than 570 °C, reduction to Fe occurs stepwise from Fe_2_O_3_ to Fe_3_O_4_ and continues to Fe (Equation (3)) [34]. The intermediate oxide, Fe_(1−*x*)_O, is not stable at temperatures lower than 570 °C. At reduction temperatures higher than 570 °C, the reduction occurs from Fe_2_O_3_ via Fe_3_O_4_ to Fe_(1−*x*)_O and continues afterward to Fe (Equation (4)):

T < 570 °C  Fe_2_O_3_ → Fe_3_O_4_ → Fe^0^ (3)

T > 570 °C  Fe_3_O_4_ → FeO → Fe^0^ (4)

In the literature, the two- (Equation (3)) or three-step reduction of Fe_2_O_3_ (Equation (5)) is discussed. The process of Fe oxide reduction is strongly dependent on various factors such as Fe content, presence of promoters/supports, the H_2_O/H_2_ ratio, catalyst preparation method, and iron precursor during reduction [35].

Fe_2_O_3_ → Fe_3_O_4_ → FeO → Fe^0^ (5)

The reduction of monometallic Fe on carbon nanotubes was studied using TPR [36]. Three main groups of hydrogen consumption peaks were referred to the multistep reduction from hematite to metallic Fe: Fe_2_O_3_ → Fe3O4→ FeO → Fe (Equation (5). The first peak was related to the reduction of Fe_2_O_3_ to Fe_3_O_4_ (~250–420 °C), the second peak was ascribed to the reduction of Fe_3_O_4_ to FeO, and the third peak was assigned to the reduction of FeO to Fe^0^ (~600–700 °C).

The authors of [37] showed that when Fe was more than 45%, reduction happened to be a three-stage process (hematite Fe_2_O_3_ → magnetite Fe_3_O_4_ → wustite FeO → Fe) (Equation (5)); however, when Fe is less than 30%, it is reduced through a two-stage process (Equation (3)). The authors of [38] suggest that reduction of iron oxides in Fe/Al_2_O_3_ occurs in a two-step process, as shown in Equation (6). The first peak at 370–390 °C represents the reduction of Fe_2_O_3_ to Fe_3_O_4_, while the second peak at 450 °C is ascribed to the reduction of Fe_3_O_4_ to FeO. The authors hypothesize that it is most likely that the strong Fe–Al_2_O_3_ interaction results in the reduction of Fe_2_O_3_ to Fe_3_O_4_, causing a shift to higher temperatures, stabilization of the FeO phase, and further suppression of the transformation of FeO to Fe on the Fe/Al_2_O_3_ catalyst.

Fe_2_O_3_ → Fe_3_O_4_ → FeO (6)

The final stage of reduction, from wüstite to metallic iron, is the slowest step and the one that constrains the whole process [39]. The XPS and TEM results showed no direct evidence of metallic elements, suggesting that the limited reducing power of gases containing high-concentration steam and CO_2_, which are weakly oxidative, is unable to further reduce the Fe^2+^ to Fe^0^ [40]. That is why the complete reduction of Fe_2_O_3_ to Fe^0^ may not occur, especially under DRM reaction conditions. It should be noted that forming FeO_x_ during dry reforming is beneficial as it reduces the accumulation of surface carbon through interactions with FeO_x_ lattice oxygen, producing CO [41].

Based on the above reasons, we suggest a two-stage reduction of both cobalt (Equation (2)) and iron (Equation (6)) oxides in 10% Co-Fe-Pt/Al_2_O_3_ catalysts as the most likely reaction.

To determine precisely which metal oxides are reduced at given temperatures is difficult due to possible peak overlaps and the identical temperature regions for the reduction of cobalt and iron oxides. We hypothesize that due to the similar temperature regions required for the reduction of Co and iron oxides, the superposition of hydrogen consumption peaks may take place. Thus, the sharp peak in the lower temperature region (so-called α-peak) in all three catalysts can likely represent the reduction of both Co_3_O_4_ to CoO and Fe_2_O_3_ to Fe_3_O_4_. The peaks are shifted to the right, in the following order: 272 °C → 286 °C → 302 °C, as Fe loadings increase in the order of 2.94 → 4.90 → 6.86 wt% (Figure 4).

In contrast, the high-temperature broad peak (so-called β-peak) shifts to the left, to the low temperature region, with increasing iron content. In this broad temperature region, both CoO and Fe_3_O_4_ may be reduced. Therefore, the second β-peaks at 406 and 427 °C may be assigned to the reduction of CoO to metallic Co and Fe_3_O_4_ to FeO for catalysts with Co/Fe ratios of 7:3 and 5:5, respectively.

For the catalyst with a higher Fe content, Co/Fe= 3:7, the third peak at 530 °C appears in the TPR profile. In this case, two separate individual peaks are observed at 386 and 530 °C, and are due to the overlap in catalysts with higher Co contents (Figure 3); hence, we suggest that these peaks can be assigned to the reduction of CoO → Co^0^ and Fe_3_O_4_ → FeO, respectively. These peaks are less intensive than the broad β-peak observed in the catalysts with higher Co contents.

In addition to the main peaks, a low-temperature shoulder at 215 and 230 °C was observed for the catalysts with higher Co contents, Co/Fe (7:3) and Co/Fe (5:5), respectively, and may be assigned to the reduction of platinum oxide. As the Fe content increases from 2.94 to 4.90, the Pt oxide reduction temperature shifts to higher temperatures of 215 → 230 °C. With a further increase in the Fe content to 6.86, the shoulder disappears, likely due to an additional shift to higher temperatures and overlapping with the Co oxide reduction peak.

Thus, the TPR-H_2_ analysis demonstrates a change in the oxidation state of Co-Fe-Pt catalysts depending on the Fe-Co ratio. The positive effect of Pt on the reducibility of Co oxides has been noted previously [24]. The present study shows that an increase in the Fe content leads to some difficulties in the reduction of the most oxidized state of Co, and this unexpectedly facilitates the reduction of the more reduced state of Co and Fe oxides. This indicates a synergistic effect due to the Co-Fe-interaction.

### 3.2. Catalyst Testing

#### 3.2.1. Dry Reforming of Methane

**Figure 5:**
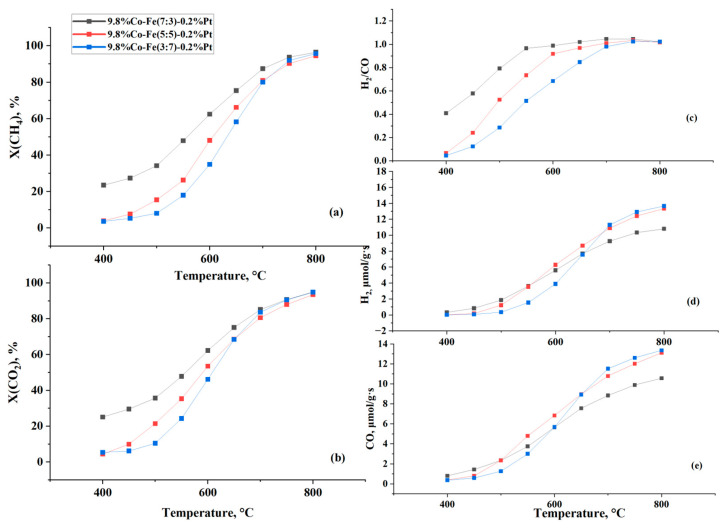
The effect of temperature on catalytic performance in DRM over the 10%Co-Fe-Pt/Al_2_O_3_ at *p* = 1 atm, CH_4_:CO_2_ = 1:1, GHSV = 1000 h^−1^: (**a**) methane conversion, (**b**) CO_2_ conversion, (**c**) H_2_/CO ratio, (**d**) hydrogen yield, and (**e**) CO yield.

Figure 5 shows the catalytic performance of all trimetallic catalysts with different Co:Fe ratios in DRM over a temperature range of 400–800 °C. The reactant conversion and product yield gradually increased with increasing temperature.

At 800 °C, the extents of conversion of all three catalysts are almost the same: X(CH_4_) = 94.6–96.4% (Figure 5a) and X(CO_2_) = 93.5–94.9% (Figure 5b). The difference in activity between catalysts with different Co:Fe ratios were observed at lower temperature; the lower the temperature, the greater the differences. Thus, the catalyst with higher Co content (ratio of Co:Fe is 7:3) is the most active; at 400° C, the extents of conversion of methane and carbon dioxide are 23.5 and 25.1%. While the catalysts with Co:Fe = 5:5 and Co:Fe = 3:7 demonstrate much lower activity: X(CH_4_) = 3.6–3.9%, X(CO_2_) = 4.4–5.4% (Figure 5a,b).

The extent of conversion and yield were compared at 700 °C to evaluate the activity of the catalysts, depending on the ratio between Co and Fe. The catalyst with a lower iron content (2.94 wt% that corresponds to Co/Fe = 7:3) outperformed the others in terms of the extent of conversions of CH_4_ and CO_2_. As Figure 5 shows, the catalyst with a Co/Fe ratio of 7:3, containing more cobalt, demonstrated better catalytic activity for the conversion of feed gases to DRM. In contrast, the catalyst with a lower iron content and a higher iron content demonstrated slightly lower reactant conversions. In other words, increasing the iron content of the catalyst leads to a decrease in methane and carbon dioxide conversion. The catalyst with a lower iron content (2.94 wt%) (Co/Fe = 7:3) outperformed the others in CH_4_ and CO_2_ conversion.

For instance, as the Fe content increased from 30 to 70%, conversions of CH_4_ and CO_2_ decreased from 87.4 to 79.7%, and from 85.2 to 83.5%, respectively. However, the opposite trend is observed in the yields of hydrogen and carbon monoxide. The H_2_ yield grows from 9.2 to 11.3 μmol/g × s while the CO yield rose from 8.8 to 11.4 μmol/g × s as iron content increased. While the decrease in conversion with increasing iron content was expected, as iron has the lowest activity among the Group 8 metals, the higher syngas yield requires an explanation. A probable cause for this may be the presence of an induction period, or so-called ‘catalyst surface development’ for the catalyst with a high iron content. High conversions of reactants, especially carbon dioxide, are observed in the initial period of the reaction, but no products are formed. Therefore, instead of describing this process as one that involves a high degree of conversion, it is more correct to say that it reflects the absorption of the initial reactants during the initial period. After saturation of the surface with adsorbed species, the catalyst reaches a steady-state regime. After that, the formation of products stabilizes, and their yield is higher due to the contribution of carbon dioxide- and methane-adsorbed species accumulated on the catalyst surface during the initial period.

However, it should be noted that the ratio of H_2_/CO slightly decreased from 1.05 to 0.98 as Fe content increased from 30% to 70%, indicating higher CO yields than H_2_. This suggests that a side reaction, such as the reverse water–gas shift reaction, which consumes H_2_, occurs simultaneously with the main reaction [22].

#### 3.2.2. Bireforming of Methane

**Figure 6:**
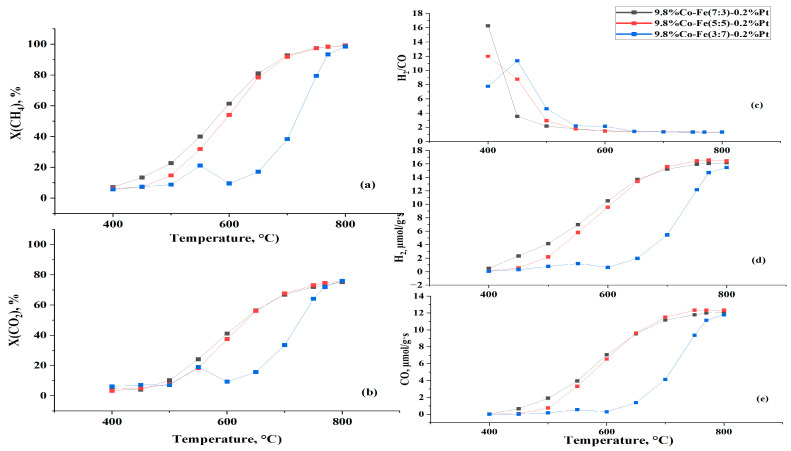
The effect of temperature on catalytic performance in BRM over the 10%Co-Fe-Pt/Al_2_O_3_ at *p* = 1 atm, CH_4_:CO_2_:H_2_O = 1:1:0.5, and GHSV = 1000 h^−1^: (**a**) methane conversion, (**b**) CO_2_ conversion, (**c**) H_2_/CO ratio, (**d**) hydrogen yield, and (**e**) CO yield.

The bireforming of methane on the Co-Fe catalysts was conducted under the following conditions: CH_4_/CO_2_/H_2_O = 1/1/0.5, T = 400–800 °C, *p* = 1 atm, and GHSV = 1000 h^−1^. Figure 6 demonstrates the temperature profiles for reactant conversions and product yields. Both profiles increase simultaneously with rising temperature due to the strong endothermic nature of the reaction. Introducing 20 vol.% steam into the DRM enhanced CH_4_ conversion but had a negative effect on CO_2_ conversion. Methane conversion was substantially higher than CO_2_ conversion, which can be attributed to the occurrence of the water–gas shift reaction (WGSR) as a side reaction that forms CO_2_ and H_2_ [42]. Consequently, as CO was consumed by the WGSR, a marginal decrease in its yield was observed upon the addition of 20 vol.% steam to the DRM.

A study by [13] investigated 5%Co/Al_2_O_3_ catalysts with different Fe contents and found that the optimal iron loading was 0.8 wt%, which yielded a H_2_/CO ratio slightly above 0.65 at 700 °C. In our study, as a result of water addition, the H_2_/CO ratio increased and ranged between 1.31 and 1.34 at 800 °C. Therefore, it follows that the introduction of water into the DRM process will positively affect the H_2_/CO ratio, allowing it to be increased and varied for further use it as a feedstock in other processes, such as Fischer–Tropsch synthesis.

It is shown that catalysts with 30 and 50% iron content demonstrated equally high catalytic activity in the BRM process. Notably, the catalyst with a 50/50 ratio of Fe/Co showed higher yields of products (H_2_, CO). The conversion of CH_4_ exceeded 97%, while the CO_2_ conversion reached 73%. This superior performance may be attributed to the optimal balance between Co and Fe sites in the 50/50 catalyst, which provides sufficient active sites for CH_4_ activation (mainly by Co) and simultaneously promotes CO_2_ dissociation and oxygen removal (enhanced by Fe) [14]. Such a synergistic interaction between Co and Fe enhances both the reforming activity and syngas yield.

**Figure 7:**
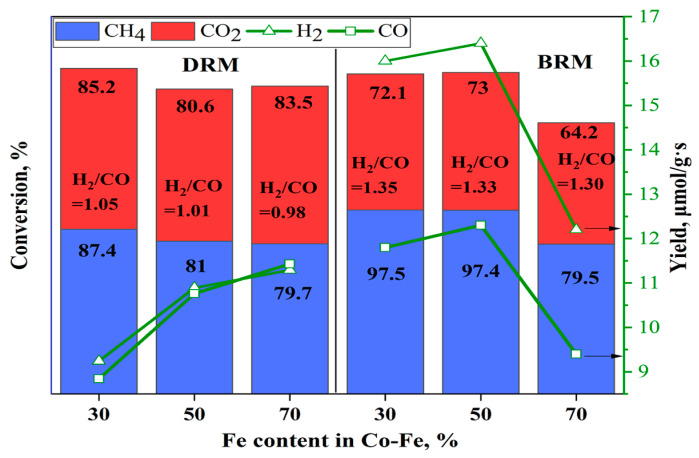
Comparison of Co-Fe-Pt catalysts with various Fe/Co ratios in DRM and BRM at T = 750 °C, 1 atm, 1000 h^−1^ (Fe contents of 30%, 50%, 70% correspond to 2.94, 4.9, 6.86 wt% of the catalyst, respectively).

A comparison of the efficiency of DRM and BRM processes over the 10%Co-Fe-Pt/Al_2_O_3_ at *p* = 1 atm, CH_4_:CO_2_:H_2_O = 1:1:0.5, GHSV = 1000 h^−1^ is presented in Figure 7. In terms of a higher hydrogen yield, the BRM process is more effective than DRM. The extent of CH_4_ conversion is substantially increased in BRM. The optimal ratio of Co/Fe in terms of higher yield of syngas is equal to 1:1. This catalyst was selected for the stability test.

**Figure 8:**
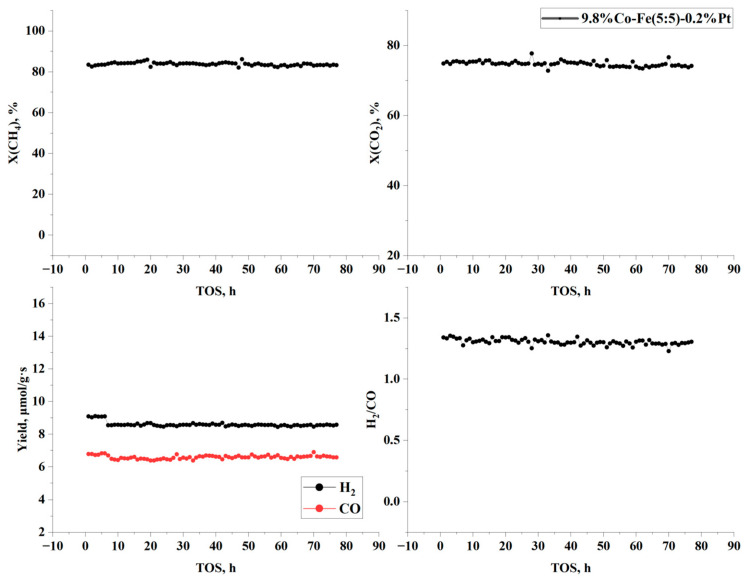
Stability test of 9.8%Co-Fe(5:5)-0.2%Pt in BRM at T = 700 °C, 1 atm, 1000 h^−1^, CH_4_/CO_2_/H_2_O = 1/1/0.5.

Stability testing was carried for the catalyst 9.8%Co-Fe(5:5)-0.2%Pt in BRM at 700 °C, 1 atm, 1000 h^−1^ for 86 h on stream (Figure 8). Initially, the profiles for the 9.8%Co-Fe(5:5)-0.2%Pt catalyst increased gradually during the first 10 h, before reaching a stable state. The catalyst exhibited stable catalytic performance and no visible coke formation.

## 4. Conclusions

The 10%Co-Fe-Pt/Al_2_O_3_ catalysts with various ratios of Co:Fe = 7:3, 5:5, and 3:7 and modified by 0.2 wt% of Pt were synthesized, characterized by a number of physicochemi-cal methods, and tested in DRM and BRM processes.

The catalysts are highly dispersed X-Ray amorphous material.

The TPR-H2 analysis demonstrates a change in the oxidation state of Co-Fe-Pt cata-lysts depending on the Fe-Co ratio. An increase in the Fe content leads to increase in the reduction temperature of the most oxidized state of Co and facilitates the reduction of the more reduced state of Co and Fe oxides. This indicates a synergistic effect due to the Co-Fe interaction. A two-stage reduction of both cobalt (Co_3_O_4_ → CoO → Co) and iron oxides (Fe_2_O_3_ → Fe_3_O_4_ → FeO) is proposed for the 10% Co-Fe-Pt/Al_2_O_3_ catalysts as the most proba-ble process. The formation of FeOx during dry reforming helps to prevent carbon deposition due to its removal by FeOx lattice oxygen, producing CO.

The synthesized Co-Fe-Pt alumina supported catalysts demonstrate the high activity and selectivity in syngas production by DRM and BRM. The extent of methane and CO_2_ conversion varied within the range of 79.5–97.5 and 64.2–85.2%, respectively, at 700–800 °C, while the H_2_/CO ratio in the resulting syngas ranged from 0.98 to 1.30, depending on the catalyst and feed composition. From the point of view of higher hydrogen yield, the BRM process is more effective than DRM. Also, the presence of water in the feed leads to de-creasing carbon accumulation on the catalyst surface due to the oxidation of surface car-bon by water with the formation of additional amounts of CO and H_2_.

Stability tests conducted for up to 80 h on stream showed no loss of activity in the 10%Co-Fe-Pt/Al_2_O_3_ catalysts in BRM. Preliminary stability tests showed stable operation for 80 h on stream. Methane was almost completely converted: X(CH_4_) = 97.4% at 750 °C.

The excellent catalytic performance of 10%Co-Fe-Pt/Al_2_O_3_ is caused by the synergetic effects of interactions within the Co-Fe-Pt system.

## Figures and Tables

**Table 1:** Comparative results for Fe-containing catalysts used in DRM.

Catalyst	Reaction Conditions	X(CH_4_), %	X(CO_2_), %	H_2_/CO	Ref.
75Ni-25Fe/Al_2_O_3_	CH_4_:CO_2_:N_2_ = 1:1:3, 600 °C	16	24	0.44	[12]
0.8Fe-5Co/Al_2_O_3_	700 °C	~50	~83	~0.65	[13]
Fe@MWCNT/Co	CH_4_:CO_2_:O_2_:He:H_2_O = 1:0.5:0.33:1.3:0.37, 800 °C, GHSV = 63,000 (mL/g.h)	46	36	0.5	[14]
Fe/MgAl_2_O_4_	CH_4_:CO_2_(1.5:1), 700 °C, GHSV = 11,706 h^−1^	79	56	-	[15]
NiAlFeO_4_ (Cl^−^)	CH_4_:CO_2_:He:Ar = 20:20:10:50, 750 °C	92	~90	~0.8	[16]
6Ni-0.2Fe/La_2_O_3_	CH_4_:CO_2_ = 1:1, WHSV = 18,000 mL h ^−1^ g_cat_^−1^, 750 °C	~70	~78	~0.92	[17]
0.5-FeNiAl	CH_4_:CO_2_ = 1:1, 700 °C	~60	~67	~0.86	[18]
0.3Fe-8Co/Al_2_O_3_	700 °C, P_CH4_/P_CO2_ = 20/20 kPa/kPa, GHSV = 36 L g_cat_^−1^ h^−1^	~68	~94	~0.8	[19]
Fe/MgAl_2_O_4_	CH_4_:CO_2_ = 1.5:1, 700 °C, *p* = 1 atm, GHSV = 11,706 h^−1^,	76.7	46.4	-	[15]
FeMo/Ni/CeO_2_-Al_2_O_3_	CH_4_:CO_2_ = 1:1, 700 °C, *p* = 1 bar, WHSV = 12,000 mL g_cat_^−1^ h^−1^	78	81	0.89	[20]
Pt/FeMo/Ni/CeO_2_-Al_2_O_3_	CH_4_:CO_2_ = 1:1, 700 °C, *p* = 1 bar, WHSV = 12,000 mL g_cat_^−1^ h^−1^	81	86	0.91	[20]

**Table 2:** BET results of Co-Fe catalysts.

Catalysts	Nominal Content, wt%			BET Surface Area, m^2^/g			Average Pore Diameter, nm		
	Co	Fe	Pt	Fresh	Spent in DRM	Spent in BRM	Fresh	Spent in DRM	Spent in BRM
9.8%Co-Fe(7:3)-0.2%Pt/Al_2_O_3_	6.86	2.94	0.2	190.8	101.3	111.7	6.8	6.0	10.3
9.8%Co-Fe(5:5)-0.2%Pt/Al_2_O_3_	4.90	4.90	0.2	168.3	103.2	95.7	6.0	7.0	7.1
9.8%Co-Fe(3:7)-0.2%Pt/Al_2_O_3_	2.94	6.86	0.2	171.9	111.7	60.6	6.3	9.7	8.1

**Table 3:** Elemental composition of the catalysts determined by EDX.

Catalyst	Samples	Co (wt%)		Fe (wt%)		Pt (wt%)		Al (wt%)		O (wt%)	
9.8%Co-Fe(7:3)-0.2%Pt/Al_2_O_3_	fresh	7.73		3.39		0		45.07		43.81	
	spent	9.13 ^a^	6.57 ^b^	3.74 ^a^	2.47 ^b^	0 ^a^	0 ^b^	51.83 ^a^	45.68 ^b^	35.30 ^a^	45.29 ^b^
9.8%Co-Fe(5:5)-0.2%Pt/Al_2_O_3_	fresh	5.21		5.05		0		44.24		45.21	
	spent	5.93 ^a^	5.90 ^b^	6.06 ^a^	5.03 ^b^	0 ^a^	0 ^b^	49.70 ^a^	46.33 ^b^	38.30 ^a^	42.64 ^b^
9.8%Co-Fe(3:7)-0.2%Pt/Al_2_O_3_	fresh	3.37		7.04		0		45.09		44.33	
	spent	3.69 ^a^	3.53 ^b^	7.65 ^a^	7.30 ^b^	0 ^a^	0 ^b^	49.58 ^a^	46.81 ^b^	39.08 ^a^	42.21 ^b^

^a^ spent in DRM ^b^ spent in BRM.

## Data Availability

The raw data supporting the conclusions of this article will be made available by the authors upon request.

## Abbreviations

The following abbreviations are used in this manuscript:

BET	Brunauer–Emmett–Teller method to measure the specific surface area and porosity
BRM	Bireforming of Methane
CCUS	Carbon Capture, Utilization, And Storage
DRM	Dry Reforming of Methane
GHSV	Gas Hourly Space Velocity
H_2_-TPR	Hydrogen Temperature-Programmed Reduction
SEM	Scanning Electron Microscopy
TEM	Transmission Electron Microscopy
X(CH_4_)	Extent of conversion of methane
X(CO_2_)	Extent of conversion of carbon dioxide
